# AG490 and PF431396 Sensitive Tyrosine Kinase Control the Population Heterogeneity of Basal STAT1 Activity in *Ube1l* Deficient Cells

**DOI:** 10.1371/journal.pone.0159453

**Published:** 2016-07-18

**Authors:** Hesung Now, Joo-Yeon Yoo

**Affiliations:** Department of Life Sciences, Pohang University of Science and Technology (POSTECH), Pohang, 37673, Republic of Korea; Wayne State University, UNITED STATES

## Abstract

A population often contains distinct sub-populations, thereby increasing the complexity of the overall heterogeneity. However, the cellular origin and biological relevance of sub-populations in cell population have not been clearly identified. Here we demonstrated the novel roles of ISGylation, which is an IFN-induced post-translational modification, controlling heterogeneity at the population level in cultured adherent cells. Without UBE1L, an E1 enzyme of ISGylation, mouse embryonic fibroblasts (MEF) exhibited low viral resistance despite high STAT1 and ISG expression compared with the wild-type MEF. We observe that *Ube1l*^*−/−*^ MEF populations consist of two behaviorally distinguishable sub-populations with distinct basal STAT1 activity, while wild-type MEF populations are unimodal. This population heterogeneity in *Ube1l* knock-out cells was perturbed by tyrosine kinase inhibitors, AG490 and PF431396. In contrast, the neutralization of type I IFN did not affect population heterogeneity. Based on these results, we concluded that UBE1L functions to adjust basal immunological states with the regulation of population heterogeneity.

## Introduction

In biological systems, most populations consist of heterogeneous sub-populations with different characteristics but not identical individuals. Classically, the heterogeneities at the genetic level such as nucleotide polymorphism, genome mutation, and chromosome instability are considered as sources of various biological phenomena, including evolution, speciation, phenotypic divergence and disease development [1–3]. At the non-genetic level, the heterogeneity in epigenetic regulations of the genome such as DNA methylation, histone modification, and chromatin structures have been also suggested as sources of various biological processes [3, 4]. In addition, intrinsic stochastic behavior of macromolecules previously considered as noise has been recently considered as a non-genetic source of heterogeneity within populations, which contributes to the diversity of cellular responses to changing environmental conditions [5, 6]. The co-existence of multiple states, independent to genetic heterogeneity, has been reported in various biological systems [7–9]. The resistant sub-populations confer survival against antibiotics or chemotherapy in bacteria or cancer cells [10, 11] and latency in human immunodeficiency virus integration [12]. During viral infection, multiple host factors, including the history of infection, cellular state of development, stages of cell cycle progression, and even the cellular morphology, are known to affect the cellular heterogeneity of host response against the virus [13]. Type I Interferon(IFN), which is the most potent anti-viral agent produced by the host, functions to disable the infected host cells, to induce the cell-intrinsic anti-viral state, and to activate the host immunity against infection [14]. The anti-viral effect of type I IFN is primarily mediated by IFN-stimulated genes (ISG), which are induced by the Janus kinase (JAK)-signal transducer and activator of transcription (STAT) pathway [15]. During anti-viral responses, the production of type I IFN is highly stochastic, since only a small fraction of virus-infected cells produce IFNs, while the infected neighboring cells go through the bystander effect [16, 17]. Different levels of signaling factors and receptors in the basal state prior to viral infection and the IFNβ-mediated feedback loop have been proposed as the source of the cellular heterogeneity that results in the stochastic IFN production, conferring viral clearance with the host survival [17, 18]. Although the consequences of cellular heterogeneity during viral infection are relatively well known, the control of population heterogeneity and the regulation of anti-viral responses are not understood.

ISGylation is a post-translational modification process that requires a cascade of enzymatic activities to conjugate IFN-stimulated gene 15 (ISG15) to target proteins [19]. The expression of ISG15, the enzymes responsible for its conjugation, and cellular target proteins such as DDX58, IRF3, PKR, and STAT1 are strongly induced by treatment of type I IFNs or viral infection [19]. These results indicate that ISGylation plays critical roles in the regulation of anti-viral immunity. However, ISGylation-deficient mice exhibit complicated phenotypes against virus infection: their susceptibility to infection by vesicular stomatitis virus and lymphocytic choriomeningitis virus is unchanged [20], while their susceptibility to infection by influenza B virus infection is increased [21, 22], compared with wild-type mice. Furthermore, the cellular targets of ISGylation are not exclusive to proteins in anti-viral responses, but include constitutive proteins with known cellular functions in cytoskeletal organization, stress responses, transcription, and even translation [23, 24]. These characteristics suggest that the function of ISGylation is neither restricted to the regulation of a single target protein nor explained by the regulation of immune signaling strength.

In this study, we demonstrated the potential role of ISGylation in the blockage of population heterogeneity to enhance anti-viral immunity. We found that two separable sub-populations with distinct basal productions of type I IFN and ISGs appear in basal *Ube1l* deficient cells. With these results, the regulation of population heterogeneity and the potential roles of ISGylation on anti-viral responses were discussed.

## Materials and Methods

### Cell, virus and reagent

Mouse embryonic fibroblasts(MEF) from wild type and *Ube1l*^*-/-*^ mice [20] (provided by Dr. K.I Kim, Sookmyung Women’s University, Korea) were maintained in Dulbecco’s modified eagle medium (DMEM) (Welgene, Korea) supplemented with 10% Fetal bovine serum (FBS) (Hyclone, Logan, UT) and 1% penicillin/streptomycin (Invitrogen, Carlsbad, CA). Influenza A virus (PR8 strain) was obtained from Dr. Adolfo Garcia-Sastre, Mount Sinai School of Medicine, USA and Sendai virus (VR-907^™^) was acquired from ATCC (Manasas, VA). For infection, cells were incubated with viruses in serum free DMEM for 1 hour and washed with PBS followed by further incubation with DMEM (10% FBS) until indicated time (including initial incubation time). Recombinant mouse IFNβ was purchased from PBL (Piscataway, NJ). Purified anti-mouse Interferon alpha/beta receptor 1 (IFNAR-1) (#127301) and mouse IgG1, κ as isotype control (#400101) used for neutralization was purchased from Biolegend (San Diego, CA). The cytokine and antibody are treated to the cells with desired concentration in DMEM (10% FBS) for indicated times. AG490 was purchase from Invivogen (San Diego, CA). SB20219, SP600125, U0126 and PD98059 were purchased from Sigma (St. Louis, MO). Jak inhibitor 1 was purchased from Merck (Kenilworth, NJ). Chemical drugs were dissolved to Dimethyl sulfoxide (DMSO) (Sigma, St. Louis, MO). DMSO was used for the control. Puromycin used for the mammalian cell selection was purchased from A.G Scientific. Inc (San Diego, CA).

### Lentiviral transduction

pMSCV-puro-HA-UBE1L was generated for lentiviral transduction of human *UBE1L*. The human *UBE1L* tagged with HA in amino-terminus from pCAGGs-HA-UBE1L[25] was polymerase chain reaction (PCR) amplified with a pair of oligonucleotides (5-gcgaattcgccaccatgtacccatacgac-3, 5-gcgaattctcacagctcatagtgcagag-3), followed by digestion with *Eco*RI. The PCR products were ligated into pMSCV-puro digested with *Eco*RI. pLL3.7-shIfnar1 was purchased (Bioneer, Korea) for lentiviral transduction of short-hairpin RNA targeting mouse specific *Ifnar1* (gcgtctacattatagatgac aa). Lentiviruses were generated and prepared as previously described [26]. Purified lentiviruses were used to treat *Ube1l*^+/+^ and *Ube1l*^-/-^ MEFs in serum-free DMEM for 12 h. After washing with phosphate buffered saline, the cells were cultured in DMEM (10% FBS, 1% penicillin/streptomycin) for 48 h. The selection process using puromycin (10 μg/ml) was performed in human *UBE1L*-transduced MEFs, followed by single colony selection and amplification. To measure the knockdown effect of *Ifnar1*, GFP-positive cells were selectively analyzed among the shIfnar1-transduced MEFs.

### FACS analysis

To measure the surface expression of H-2Kb, cells were washed with fluorescence associated cell sorter (FACS) buffer (1% FBS in phosphate buffered saline), followed by staining with anti-H-2Kb conjugated with FITC or PE (#553569 or #553570, BD Pharmingen, San Diego, CA). To measure the total internal expression of H-2Kb, cells were fixed with 4% paraformaldehyde and permeabilized with 0.1% saponin (Sigma) in FACS buffer. Fixed and permeabilized cells were stained with anti-H-2Kb conjugated with FITC or PE. Stained cells were washed with FACS buffer and analyzed with a flow cytometer (FACSCalibur^™^, BD Biosciences, San Jose, CA). To sort cells according to H-2Kb expression levels, a flow cytometer associated with a cell sorting system (FACSAria^™^, BD Bioscience; Moflo^™^ XDP, Beckman Coulter, Brea, CA) was used.

### Total RNA preparation, real time quantitative and semi-quantitative PCR

Total RNAs were prepared using RNAiso plus (TAKARA, Japan). 500ng of RNA were reverse transcribed with oligo-dT (for cellular genes) or random hexamer (for viral genes) using ImProm-II Reverse transcriptase system (Promega, Madison, WI). Quantitative realtime PCR of cDNA were performed with StepOne Plus Real-Time PCR system (Applied Biosystems, Foster City, CA). Using the same cDNA acquired from reverse transcripation reaction, routine semi-quantitative PCR was performed and analyzed by agarose gel electrophoresis with EtBr staining. Sequence information of oligonucleotide used for this study is provided in S1 Table.

### Immunoblot analysis

Cells were lysed with lysis buffer (150 mM NaCl, 25 mM Tris–HCl, pH 7.4, 1% Triton X-100, 0.5% Deoxycholic acid, 0.1% SDS) supplemented with protease inhibitors and phosphatase inhibitors. Total lysates (10-30ug) were separated on SDS-PAGE and transferred to nitrocellulose membranes. The expressions of interested proteins were analyzed using specific antibodies according to manufacturer’s protocol. Antibodies used for immunoblot analysis were listed in S2 Table.

## Results

### *Ube1l*^*−/−*^ MEFs basally express high levels of anti-viral genes but exhibit low viral resistance

**Fig 1:**
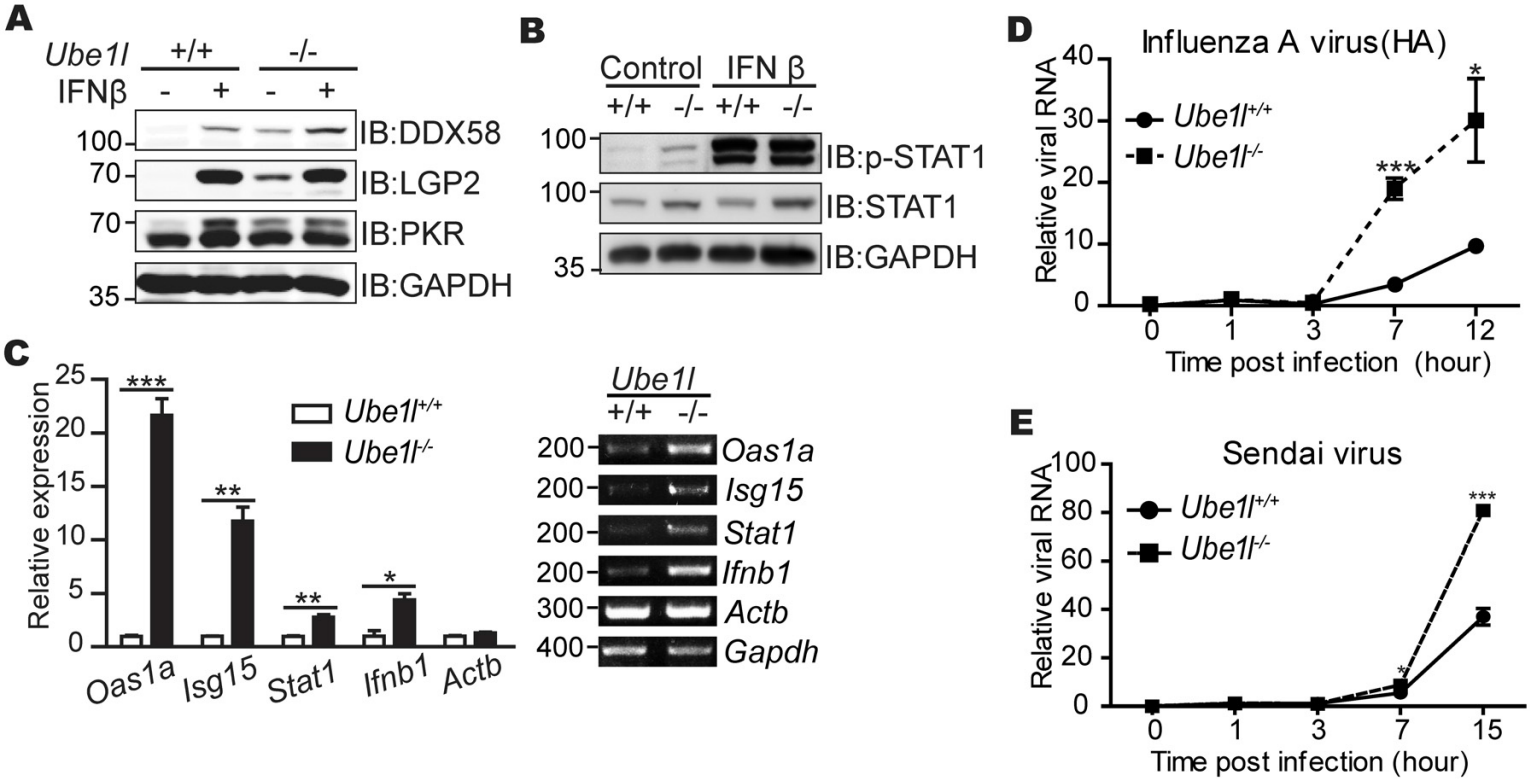
The *Ube1l*-deficient MEFs are more susceptible to viruses with higher STAT1 activity. (A) Immunoblot assay for measuring expression of indicated genes in *Ube1l*^*+/+*^ and *Ube1l*^*-/-*^ MEF with or without IFNβ (100 U/ml, 16 hour). (B) Immunoblot assay for measuring STAT1 activity (phosphorylation of Tyr701 on STAT1) in in *Ube1l*^*+/+*^ and *Ube1l*^*-/-*^ MEFs with or without IFNβ (100 U/ml, 30 min). (C) Relative mRNA expression of the indicated genes in basal *Ube1l*^*+/+*^ and *Ube1l*^*-/-*^ MEFs. *Left*–Using real-time quantitative PCR, normalizing with *Gapdh*. *Right*–Representative agarose gel image using conventional reverse-transcription PCR. Numbers beside image indicate the size of PCR product (base pairs). (D) Intracellular viral RNA normalizing with *Gapdh* in *Ube1l*^*+/+*^ and *Ube1l*^*-/-*^ MEFs infected with influenza A virus (10 TCID50) for the indicated time. (E) Relative amounts of intracellular viral RNA normalizing with *Gapdh* in *Ube1l*^+/+^ and *Ube1l*^*-/-*^ MEFs infected with Sendai virus (10 TCID50) for indicated time. (A-B) Numbers beside blot images indicate the molecular weight (kDa). (C-E) Mean and standard error obtained from three independent experiments, unpaired t test, * p<0.05, ** p<0.01, ***p<0.001.

UBE1L is an E1 enzyme that is required for the conjugation of ISG15 to target proteins [20]. To understand the effects of ISGylation in the anti-viral response, we first measured the expression of various genes required for anti-viral responses in *Ube1l*^***−/−***^ mouse embryonic fibroblasts (MEFs). As previously reported [20], *Ube1l*^***−/−***^ MEF did not show any significant defect in anti-viral protein expressions such as DDX58, LGP2, and PKR upon IFNβ treatment (Fig 1A). Consistent with that, IFNβ-mediated activation of STAT1 (phosphorylation at Tyr 701 residue) which is required for ISG expressions was also not significantly altered in *Ube1l*^*−/−*^ MEFs (Fig 1B). These results indicate that UBE1L does not play any significant regulatory roles in type I IFN signaling pathways. However, the effects of ISGylation deficiency were mainly exerted in the basal state. ISG expression and the STAT1 phosphorylation of the basal *Ube1l*^***−/−***^ MEF were significantly higher than that of the wild-type cells. In the basal condition, mRNA expression of various ISGs, including *Oas1a*, *Isg15*, and *Stat1*, was significantly increased in ISGylation-deficient *Ube1l*^***−/−***^ MEFs (Fig 1C); the same was observed for *Ifnb1*. Despite higher levels of basal STAT1 activity and anti-viral ISG expression, *Ube1l*^***−/−***^ cells were more susceptible to viral infection (Fig 1D and 1E). These results suggest that the functions of UBE1L protein cannot simply explained by the overall expression levels of anti-viral genes in the cellular population.

### *Ube1l* deficient cells generate two sub-populations of distinct STAT1 activity

**Fig 2:**
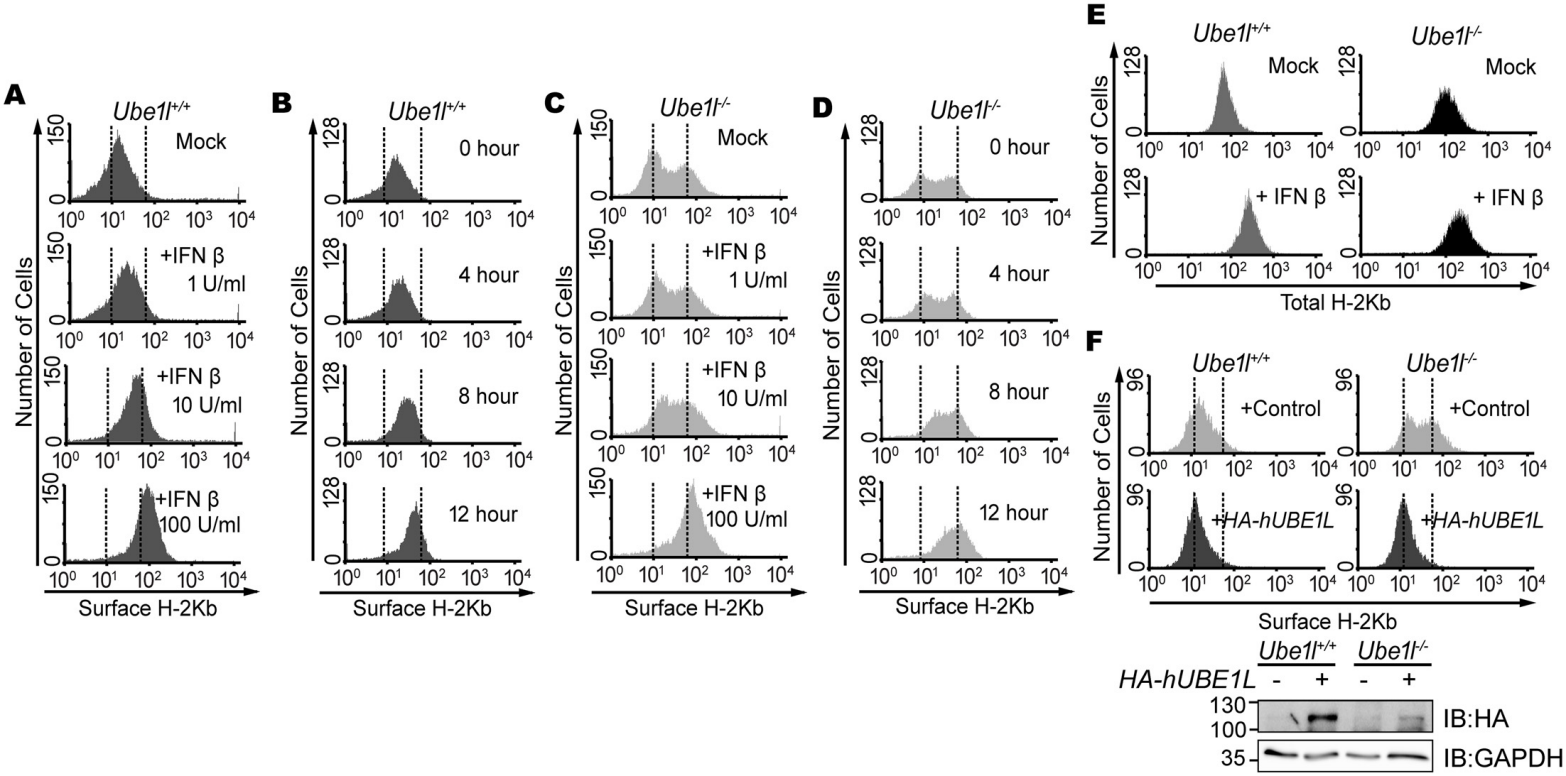
*Ube1l*-deficient population harbors sub-populations with distinct surface H-2Kb expression. (A) FACS analysis of surface H-2Kb expression on *Ube1l*^*+/+*^ MEFs with indicated amount of IFNβ for 16hour. (B) FACS analysis of surface H-2Kb expression on *Ube1l*^*+/+*^ and MEFs, with 100U/ml of IFNβ for indicated time. (C) FACS analysis of surface H-2Kb expression on *Ube1l*^*-/-*^ MEFs with indicated amount of IFNβ for 16hour. (D) FACS analysis of surface H-2Kb expression on *Ube1l*^*-/-*^ and MEFs, with 100U/ml of IFNβ for indicated time. (E) With or without the treatment of IFNβ (100U/ml, 16 h), *Ube1l*^*+/+*^ and *Ube1l*^*-/-*^ MEFs were harvested and permeabilized followed by FACS analysis for the whole cell H-2Kb expression. (F) *Top*—FACS analysis of surface H-2Kb expression on *Ube1l*^*+/+*^ and *Ube1l*^*-/-*^ MEFs transduced with the control or *HA-hUBE1L*-lentivirus. *Bottom*—Immunoblot for confirming HA-UBE1L expression. Numbers beside blot images indicate the molecular weight (kDa).

Because the total STAT1 activity was not correlated with the cellular responses against viral infection, we examined the heterogeneity of basal STAT1 activity within the whole population. Therefore, we monitored the cell-cell variations of STAT1 activity by measuring the surface presentation of class I MHC (H-2Kb). Its surface presentation is known to be induced by activated STAT1 [27]. In the population of *Ube1l*^*+/+*^ cells, the surface H-2Kb expression was homogeneous, with unimodal distributions (Fig 2A and 2B). Upon stimulation with IFNβ, the whole population of the *Ube1l*^*+/+*^ MEFs shifted to the right, indicating that surface H-2Kb expression was uniformly enhanced in this cell type. While the surface H-2Kb expression observed in *Ube1l*^*+/+*^ cells was unimodal, in *Ube1l*^***−/−***^ cells, it was bimodal and heterogeneous with one sub-population showing low surface H-2Kb and another showing high H-2Kb expression (Fig 2C and 2D). Upon stimulation with IFNβ, the H-2Kb^low^ sub-population shifted to the right and merged to the H-2Kb^high^ sub-population. Meanwhile, the total intracellular H-2Kb protein levels were unimodal in both *Ube1l*^*+/+*^ and *Ube1l*^***−/−***^ cells (Fig 2E), indicating that the bimodal surface presentation of H-2Kb does not happen due to changes in the steps of H-2Kb production. To examine whether the segregation of sub-population observed in the *Ube1l*^***−/−***^ cells was caused by the lack of UBE1L, a lentiviral vector containing *HA-hUBE1L* was transduced into the *Ube1l*^-/-^ cells (Fig 2F). This reconstitution abrogated the bimodality of *Ube1l*^***−/−***^ cells, resulting in homogeneous surface H-2Kb expression, confirming that UBE1L is responsible for population segregation.

**Fig 3:**
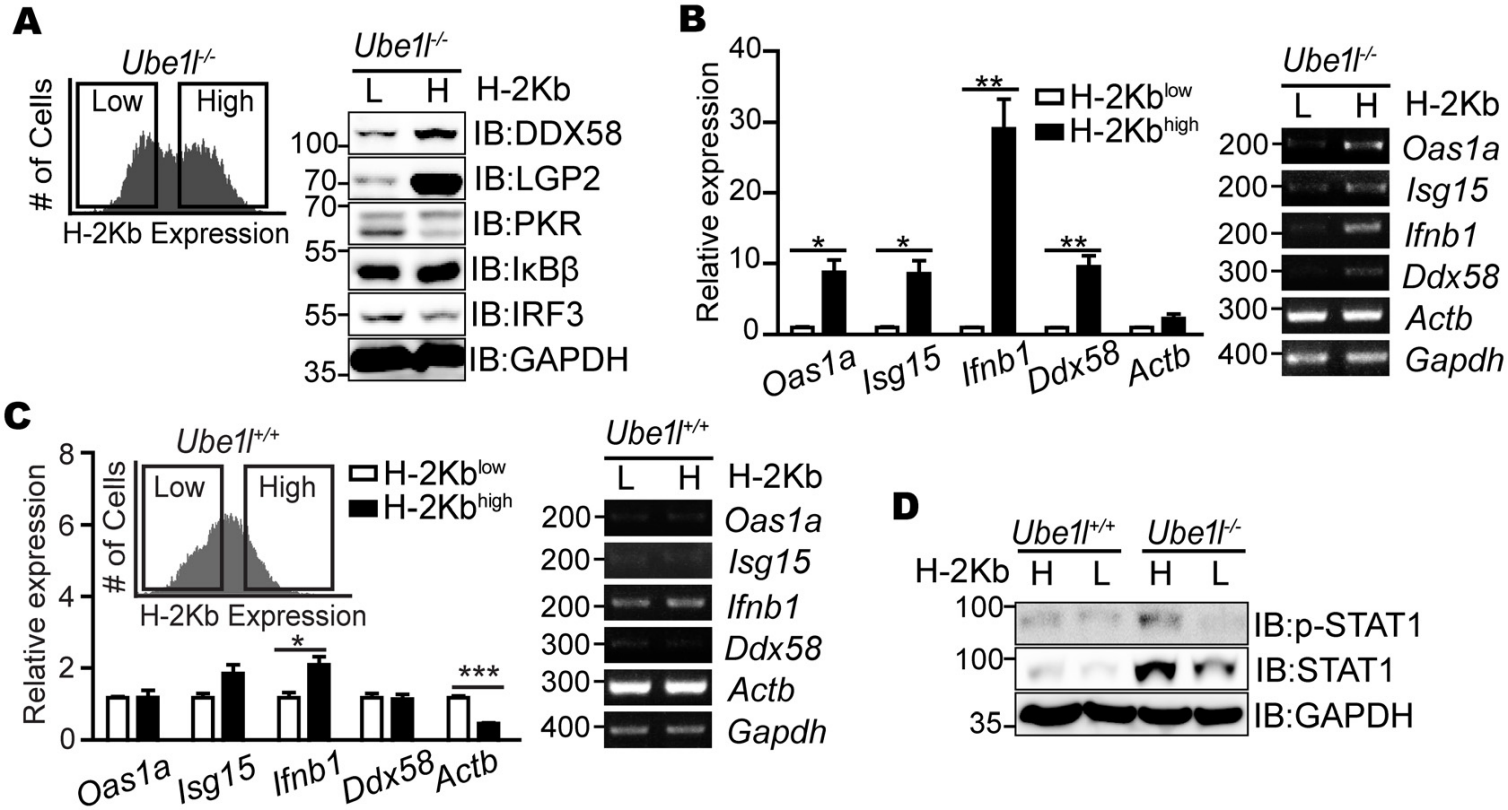
Two sub-populations of *Ube1l*^*-/-*^ MEFs possesses distinct basal STAT1 activity. (A) The *Ube1l*^*-/-*^ sub-populations with high and low H-2Kb expression (H and L, respectively) were sorted using FACS and subjected to immunoblot assays with indicated antibodies. (B) Relative mRNA expression of the indicated genes in the *Ube1l*^*-/-*^ sorted according to H-2Kb expression. (C) *Ube1l*^*+/+*^ MEF were sorted using the intensity value that separates H-2Kb^high^ and H-2Kb^low^ *Ube1l*^-/-^, and relative mRNA expression of the indicated genes were measured. (D) Immunoblot assay for measuring basal STAT1 activity of *Ube1l*^*+/+*^ and *Ube1l*^*-/-*^ MEFs sorted separately according to surface H-2Kb expression. (A, D) Numbers beside blot images indicate the molecular weight (kDa). (B, C) *Left*–Using real-time quantitative PCR, normalizing with *Gapdh*. Mean and standard error obtained from three independent experiments, unpaired t test, * p<0.05, ** p<0.01, ***p<0.001. *Right*–Representative agarose gel image using conventional reverse-transcription PCR. Numbers beside image indicate the size of PCR product (base pairs).

To determine whether the *Ube1l*^***−/−***^ sub-populations with distinct surface H-2Kb expression exhibit distinct STAT1 activity, each sub-population was separately sorted according to H-2Kb expression (H-2Kb^high^ and H-2Kb^low^) with FACS, and expression of various ISGs was measured (Fig 3A and 3B). Consistent with the results for surface H-2Kb, the H-2Kb^high^ cells expressed higher levels of ISGs compared to the H-2Kb^low^ cells. Using the mean intensity value to separate the sub-populations of *Ube1l*^***−/−***^ cells, we also artificially sorted *Ube1l*^*+/+*^ cells and measured ISGs (Fig 3C). Unlike *Ube1l*^***−/−***^ cells, significant differences in ISG expression were not observed in wild-type cells. Consistent with ISG and *Ifnb1* expression, STAT1 phosphorylation levels in the sorted *Ube1l*^***−/−***^ MEF were revealed to be different with higher STAT1 phosphorylation on H-2Kb^high^ than on H-2Kb^low^, while no significant difference was observed in sorted *Ube1l*^*+/+*^ MEF (Fig 3D). Based on these findings, we conclude that the UBE1L functions to control population segregation based on its differential basal STAT1 activity and ISG expressions.

### Bimodal STAT1 activity is not mediated by basal type I IFN signaling

**Fig 4:**
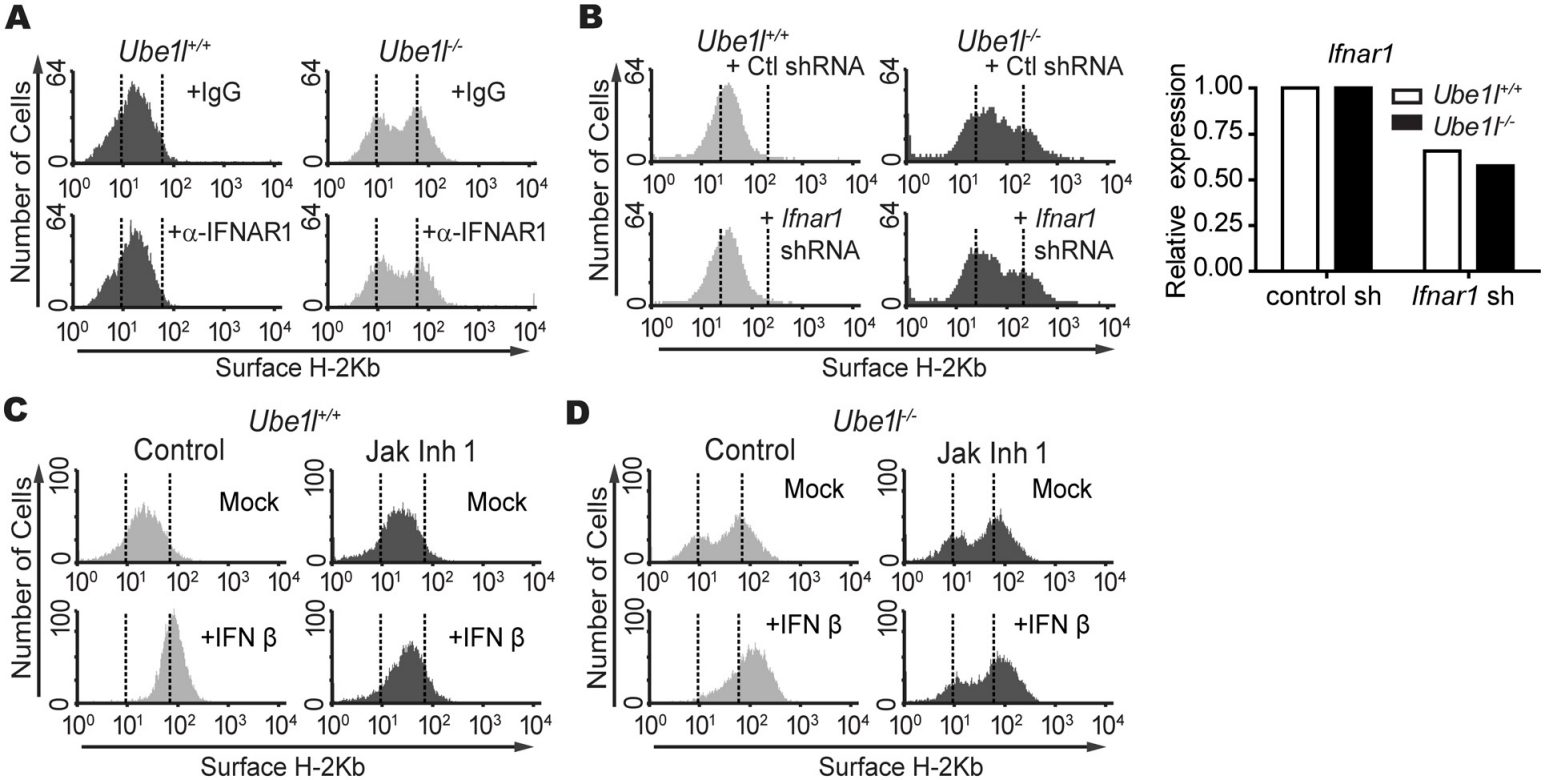
Bimodality in *Ube1l* deficient MEF populations are not mediated by basal type I IFN signaling. (A) FACS analysis of surface H-2Kb expression on MEFs treated with a neutralizing antibody for IFNAR1 (5 μg/ml) or the isotype control. (B) *Left—*FACS analysis of surface H-2Kb expression in MEFs transduced with the control or mouse *Ifnar1* shRNA lentivirus. *Right–*Relative expression of *Ifnar1* mRNA normalizing with *Gapdh* in control or *Ifnar1* shRNA lentivirus treated MEF. (C-D) *Ube1l*^*+/+*^ (C) and *Ube1l*^*-/-*^ (D) were treated with 10uM of Jak Inhibitor 1 (Jak Inh 1) for 4 hours followed by additional 12 hours of IFNβ (100U/ml) treatment. Samples were harvested and measure the H-2Kb expression with FACS.

In search for signals that regulate basal STAT1 activity *in Ube1l*^***−/−***^ cells, we first examined the effect of basal Type I IFN. Type I IFN is a major agonist for STAT1 activation and ISG expression during the viral infection [14, 15]. Furthermore, significantly different amounts of basal *Ifnb1* mRNA expressions were detected between two sub-population of *Ube1l*^***−/−***^ MEF (Fig 3C). To test this possibility, the neutralizing antibody of type I IFN receptor (IFNAR1) was treated to block the basal type I IFN signaling activity and the population heterogeneity was measured (Fig 4A). Despite the neutralization, two sub-populations were still present in *Ube1l*^***−/−***^ MEFs. The inhibition of type I IFN signaling activities with the lentiviral transduction of shRNA specific for mouse *Ifnar1* also did not alter the bimodality of *Ube1l*^***−/−***^ MEFs (Fig 4B). Consistent with these results, the inhibition of JAK activities with the chemical inhibitor (Jak Inhibitor I) did not alter the bimodality in *Ube1l*^***−/−***^ MEFs, although with complete inhibition of type I IFN signaling (Fig 4C and 4D). These results imply that the bimodality of basal STAT1 activity in *Ube1l*^***−/−***^ MEFs are not mediated by type I IFN or type I IFN-mediated signaling pathways.

### Kinase inhibitors, AG490 and PF431396, abrogate bimodality of basal STAT1 in *Ube1l*^*−/−*^ MEFs

**Fig 5:**
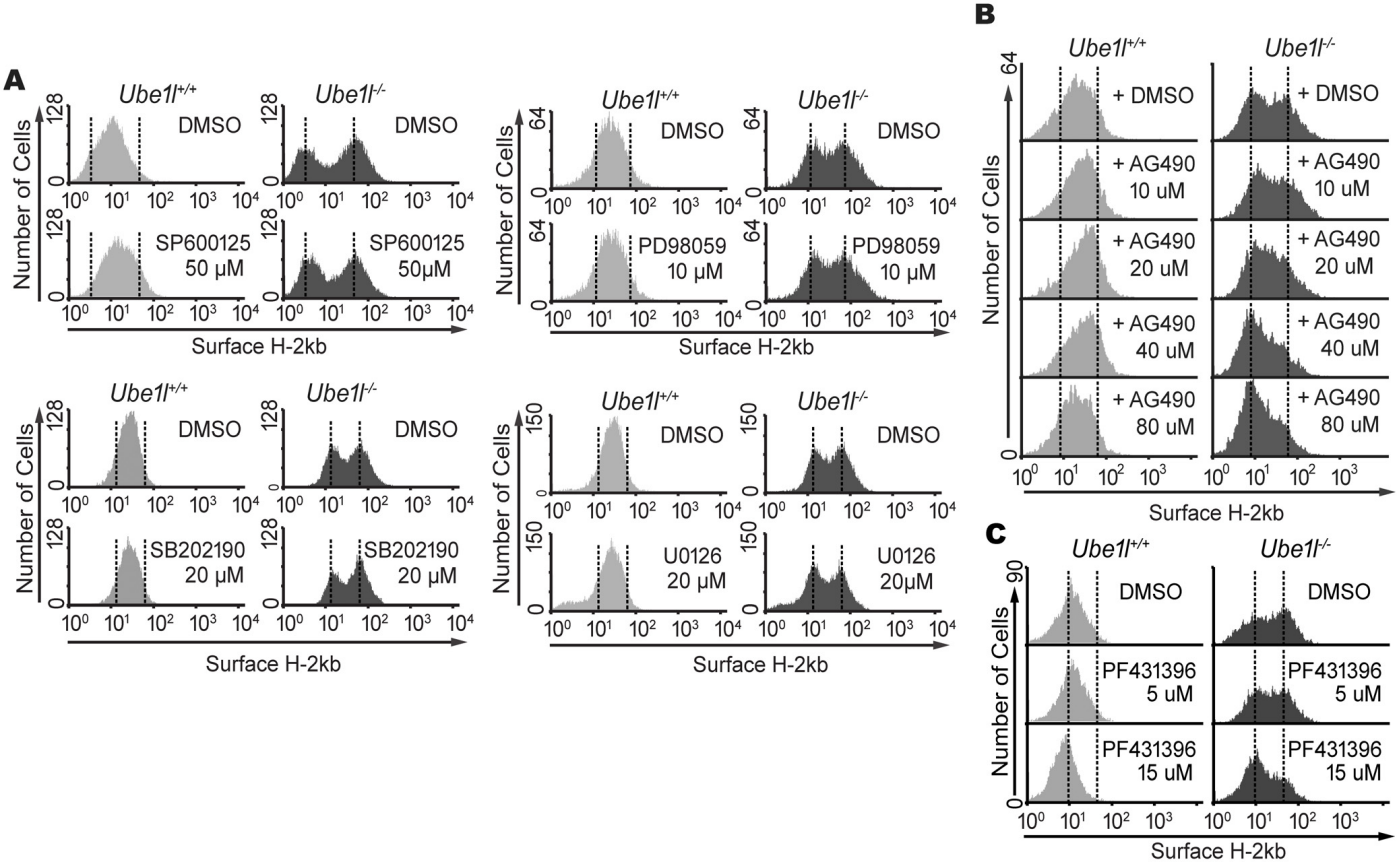
AG490 and PF431396 block the bimodality in basal surface H-2Kb expression of *Ube1l* deficient MEFs. (A) *Ube1l*^*+/+*^ and *Ube1l*^*-/-*^ MEF were treated with indicated chemical inhibitor (16hour, indicated amount), and were analyzed by FACS for surface H-2Kb expression. SP600125 is JNK inhibitor, SB202190 is p38 inhibitor, and both PD98059 and U0126 are MEK1/2 inhibitors. (B) Surface H2Kb expression in MEFs treated with AG490 (16 h, indicated amount). (C) Surface H2Kb expression in MEFs treated with PF431396 (16 h, indicated amount).

To elucidate the intracellular signaling responsible for the maintenance of bimodality of *Ube1l*^***−/−***^ MEF, we examined other kinases that are known to affect STAT1 activity. Therefore, we first tested the effect of mitogen-activated protein kinases, using SP600125 (inhibitor of JNK), SP202190 (inhibitor of p38), PD98059 (inhibitor of MEK1/2), or U0126 (inhibitor of MEK1/2). However, none of these chemical inhibitors exhibited a significant change in the bimodality of basal surface H-2Kb expression in *Ube1l*^***−/−***^ MEF (Fig 5A). In contrast, AG490, tyrosine kinase inhibitor known to inhibit epidermal growth factor receptor (EGFR), in addition to JAK2, abrogated the bimodality of basal H-2Kb expression (Fig 5B). While the *Ube1l*^*+/+*^ cells were not significantly affected, AG490 treatment led the H-2Kb^high^ group to merge with the H-2Kb^low^ group in *Ube1l*^***−/−***^ cells. Similarly, PF431396, a kinase inhibitor known to inhibit FAK1 and PYK2, affected the population heterogeneity (Fig 5C). Upon PF431396 treatment, the H-2Kb^high^ sub-population shifted to the H-2Kb^low^ group in *Ube1l*^***−/−***^ MEFs.

**Fig 6:**
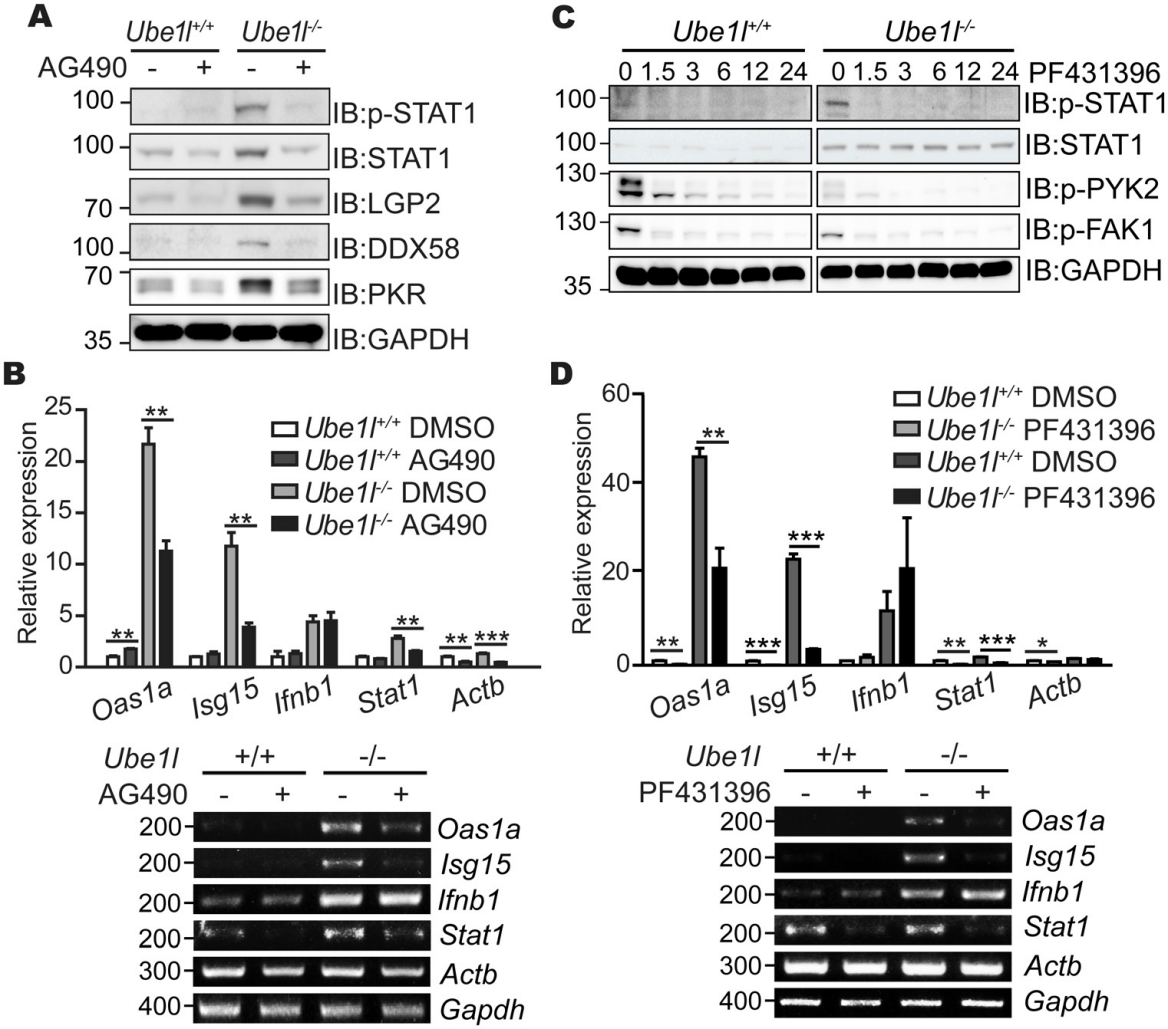
Effect of AG490 and PF431396 inhibitor on basal STAT1 activity and ISG expression. (A) Immunoblot assay for measuring basal STAT1 activity and expression of indicated genes in MEFs treated with AG490 (50 μM, 16 h). (B) Relative mRNA expression of the indicated genes were measured in MEFs treated with AG490 (50 μM, 16hr). (C) Immunoblot assay for measuring basal STAT1 activity and expression of indicated genes in MEFs treated with PF431396 (10 μM, indicated time). Numbers above the blot denotes treatment time in hours. (D) Relative mRNA expression of the indicated genes were measured in MEFs treated with PF431396 (15 μM, 12 h). (A, C) Numbers beside blot images indicate the molecular weight (kDa). (B, D) *Top*–Using real-time quantitative PCR, normalizing with *Gapdh*. Mean and standard error obtained from three independent experiments, unpaired t test, * p<0.05, ** p<0.01, ***p<0.001. *Bottom*–Representative agarose gel image using conventional reverse-transcription PCR. Numbers beside image indicate the size of PCR product (base pairs).

To address whether AG490-sensitive kinase activity controls the basal STAT1 activity observed in *Ube1l*^***−/−***^ MEFs, we measured the basal phosphorylation of STAT1 (Tyr701) in *Ube1l*^***−/−-/-***^ MEFs with the AG490 treatment (Fig 6A). Active STAT1 was strongly reduced in the AG490-treated *Ube1l*^***−/−***^ cells, resulting similar levels of STAT1 phosphorylation observed in *Ube1l*^*+/+*^. Consistent with that, the protein expressions of various anti-viral ISGs, including STAT1 itself, were significantly reduced in the same experimental samples. AG490 also significantly reduced mRNA expression of various ISGs in *Ube1l*^***−/−***^ cells (Fig 6B). However, expression of *Ifnb1* mRNA was not significantly altered by AG490 treatment, suggesting that STAT1 bimodality is not responsible for the type I IFN synthesis.

Similar to AG490, PF431396-sensitive kinase also affected the basal STAT1 activity in *Ube1l*^***−/−***^ MEFs (Fig 6C). We measured the basal activity of STAT1 after PF431396 treatment. As the phosphorylation of FAK1 (Tyr397) and PYK2 (Tyr402) were reduced by PF431396 treatment, phosphorylated form of STAT1 was also reduced. Furthermore, FAK inhibition also lowered the expression levels of ISGs, but not of *Ifnb1* (Fig 6D). From these results, we conclude that the cellular tyrosine kinases affected by AG490 and PF431396 inhibitors are responsible for the bimodality of basal STAT1 activity and ISG expression in *Ube1l*-deficient cells.

## Discussion

Our data suggest that UBE1L functions to prevent population heterogeneity, which originates from the bimodal STAT1 activity. Using the ISGylation-deficient *Ube1l*^***−/−***^ cells, we found that UBE1L suppresses basal STAT1 activity in the overall population of the cells, but also increases the resistance against the viral infection. During research to solve this contradiction, it was revealed that *Ube1l*^***−/−***^ MEFs populations consisted of two sub-populations with distinct basal STAT1 activities. This population segregation was revealed to be originated from the basal kinase activity sensitive to AG490 and PF431396, but not from the constitutive type I IFN activity present in the basal states.

In the present study, the basal signals that resulted in the bimodality of STAT1 in *Ube1l*^***−/−***^ MEF are still elusive. Considering the result of the use of a neutralizing antibody and shRNA against type I IFN receptor, we excluded the involvement of type I IFN in this process. We also excluded the involvement of type II and type III IFNs in population segregation of the *Ube1l*^***−/−***^ MEFs, because Jak Inhibitor 1 that is known to inhibit JAK1, JAK2, and TYK1, which are involved in IFN signaling, did not show any significant effect [28–30]. Instead, we propose that the tyrosine kinases sensitive to AG490 or PF431396 is responsible for the bimodality of STAT1 in *Ube1l*^***−/−***^ MEFs. While AG490 targets various kinases, including JAK2, EGFR, and ERBB2 [31, 32], PF431396 targets for both FAK1 and PYK2 [33], which are members of the FAK family. Based on the results acquired with the pan-JAK inhibitor (Jak Inhibitor 1), by excluding JAK2, we suspected that EGFR, ERBB2, FAK1 or Pyk2 were candidate kinases responsible for the basal STAT1 activity. The association of EGFR and FAK signaling pathway has been previously reported [34, 35]. During the growth factor-induced cell migration or adhesion, EGFR is known to associate with the integrin receptors and form multi-protein signaling complexes [36]. Both FAK and STAT1 are reported to be found in this complex [35, 37–39]. Therefore, it is logical to consider basal growth factor or cell adhesion signaling activities as potential sources of bimodality observed in *Ube1l*-deficient cells.

The basal population heterogeneity that we observed in *Ube1l*-deficient MEFs appeared in the conventional culture method. However, in physiological circumstances, various environmental stresses are persistently applied to the cellular population [40, 41]. These environmental factors, working as selection pressures, determine population dynamics with multiple levels of non-genetic and genetic heterogeneity [3, 42–44]. Although we applied the type I IFN or chemical inhibitors and observed the change of population composition of MEFs, the effects of prolonged stresses were not considered in this research. Considering the selection effect of long-term persisting stresses on the biological systems, including cancer development [3, 42], further researches monitoring population dynamics upon prolonged stresses are required for identifying the cause and the physiological meaning of cellular heterogeneity. In those experiments, the dynamics of population originated from genetic heterogeneity should be also considered.

In this study, we presented the effect of population heterogeneity in the regulation of anti-viral signaling. Beneficial or detrimental outcomes of population heterogeneity may depend on the nature of the changing environment or selection pressures [45]. In a harsh environment with restricted resources, increasing heterogeneity with multiple sub-populations may confer benefits of cooperation with the reduction of intra-population competition for the overall fitness of the entire population [46, 47]. Meanwhile, reducing heterogeneity restricts individuals to extreme behaviors, which are wasteful in a moderate environment [45]. The disruption of population heterogeneity regulation frequently results in disease progression, particularly cancer [3, 11, 48]. During the stepwise development of the majority of cancer, genetic heterogeneity is highly increased by chromosome instability with non-clonal chromosome aberration required for the progression to the next stage [42]. In addition to that, phenotypic diversification originated from non-genetic heterogeneity is also observed in a cancer cell population, resulting in the occurrence of drug resistant sub-populations [11]. These results imply that the regulation of population heterogeneity is essential to maintain the homeostasis of biological systems. However, in host-virus interaction, the physiological meaning of population heterogeneity has not been extensively studied. Although the working mechanism of how the heterogeneity affects host anti-viral immunity remained to be answered, our study suggests the potential roles of ISGylation on the host anti-viral response with the regulation of population heterogeneity.

## Supporting Information

S1 Table Oligonucleotide used for real-time quantitative PCR and conventional RT PCR. (DOCX)

S2 Table Antibodies used for immunoblot assay. (DOCX)
