# Supplementary material for: AG490 and PF431396 Sensitive Tyrosine Kinase Control the Population Heterogeneity of Basal STAT1 Activity in Ube1l Deficient Cells
Source: PLoS One. 2016 Jul 18;11(7):e0159453. doi: 10.1371/journal.pone.0159453 (PMC4948911; doi:10.1371/journal.pone.0159453)
Supplement: S1 Table — (DOCX) [file pone.0159453.s001.docx]

**S1 Table-** **Oligonucleotide used for real-time quantitative PCR and conventional RT PCR**

| **Targets** | **Sequence of oligonucleotide** | |
| --- | --- | --- |
|  | **Forward primer** | **Reverse primer** |
| ***Oas1a* (Mouse)**  **NM_145211.2** | 5-GCAGCGCCCAACCAAGCT-3 | 5-CCAGTTCCAAGACGGTCC-3 |
| ***Isg15* (Mouse)**  **NM_015783.3** | 5-CCTCTGAGCATCCTGGT-3 | 5-AGGCCGTACTCCCCCAG-3 |
| **Stat1 (Mouse)**  **NM_001205313.1**  **NM_009283.4**  **NM_002305314.1** | 5-CAATATTGACAAAGACCACG-3 | 5-CCGGGACATCTCATCAAAC-3 |
| ***Ifnb1* (Mouse)**  **NM_010510.1** | 5-CAGCACTGGGTGGAATGAGAC-3 | 5-TCCTGAAGATCTCTGCTCGGAC-3 |
| ***Ddx58* (Mouse)**  **NM_172689.3** | 5-TCCCAGCAATGAGAATCCT-3 | 5- GTCAATGCCTTCATCAGC-3 |
| ***Actb* (Mouse)**  **NM_007393.5** | 5-TCATGAAGTGTGACGTTGACATCCGT-3 | 5-CCTAGAAGCATTTGCGGTGCACGATG-3 |
| ***Gapdh* (Mouse)**  **NM_001289726.1**  **NM_008084.3** | 5-AACTTTGGCATTGTGGAAGGGCTC-3 | 5-TGGAAGAGTGGGAGTTGCTGTTGA-3 |
| ***Ifnar1*(Mouse)**  **NM_010508.1** | 5-GCTGTGTGAGAAAACACGTCCAGG-3 | 5-CAATACTGCGGGGAGGCTTGAG-3 |
| **HA(Influenza A virus)** | 5-TTGCTAAAACCCGGAGACAC-3 | 5-CCTGACGTATTTTGGGCACT-3 |
| **Sendai virus genome** | 5-GACGCGAGTTATGTGTTTGC-3 | 5-TTCCACGCTCTCTTGGATCT-3 |
