# Supplementary material for: AG490 and PF431396 Sensitive Tyrosine Kinase Control the Population Heterogeneity of Basal STAT1 Activity in Ube1l Deficient Cells
Source: PLoS One. 2016 Jul 18;11(7):e0159453. doi: 10.1371/journal.pone.0159453 (PMC4948911; doi:10.1371/journal.pone.0159453)
Supplement: S2 Table — (DOCX) [file pone.0159453.s002.docx]

**S2 Table - Antibodies used for immunoblot assay**

| **Target** | **Material information** | |
| --- | --- | --- |
|  | **Provider** | **Catalogue number** |
| **p-STAT1(Y701)** | Cell Signaling | 9171L |
| **p-STAT1(Y701)** | Cell Signaling | 7649 |
| **LGP2** | PTG Lab | 11355-1-AP |
| **DDX58** | Santa Cruz Biotechnology | SC-48929 |
| **STAT1 p91** | Santa Cruz Biotechnology | SC-417 |
| **PKR** | Santa Cruz Biotechnology | SC-708 |
| **IκBβ** | Santa Cruz Biotechnology | SC-945 |
| **IRF3** | Santa Cruz Biotechnology | SC-9082 |
| **p-PYK2(Y402)** | Santa Cruz Biotechnology | SC-101790 |
| **p-FAK(Y397)** | Santa Cruz Biotechnology | SC-11765-R |
| **HA** | Roche | 1153816001 |
| **GAPDH** | Chemicon | MAB347 |
